# Gamma Oscillations as a Neural Signature of Shifting Times in Narrative Language

**DOI:** 10.1371/journal.pone.0121146

**Published:** 2015-04-13

**Authors:** Sanne Gøren Brederoo, Laura Simone Bos, Olga Dragoy, Roelien Bastiaanse, Giosuè Baggio

**Affiliations:** 1 Center for Language and Cognition, University of Groningen, Groningen, Netherlands; 2 NeuroImaging Center, University Medical Center Groningen, Groningen, Netherlands; 3 National Research University Higher School of Economics, Moscow, Russia; 4 Brain and Language Lab, International School for Advanced Studies, Trieste, Italy; 5 Language Acquisition and Language Processing Lab, Department of Language and Literature, Norwegian University of Science and Technology, Trondheim, Norway; ARC Centre of Excellence in Cognition and its Disorders (CCD), AUSTRALIA

## Abstract

Verbs and other temporal expressions allow speakers to specify the location of events in time, as well as to move back and forth in time, shifting in a narrative between past, present and future. The referential flexibility of temporal expressions is well understood in linguistics but its neurocognitive bases remain unknown. We aimed at obtaining a neural signature of shifting times in narrative language. We recorded and analyzed event-related brain potentials (ERPs) and oscillatory responses to the adverb ‘now’ and to the second main verb in Punctual (‘An hour ago the boy stole a candy and *now* he *peeled* the fruit’) and Iterative (‘The entire afternoon the boy stole candy and *now* he *peeled* the fruit’) contexts. ‘An hour ago’ introduces a time frame that lies entirely in the past, ‘now’ shifts the narrative to the present, and ‘peeled’ shifts it back to the past. These two referential shifts in Punctual contexts are expected to leave very similar traces on neural responses. In contrast, ‘The entire afternoon’ specifies a time frame that may encompass past, present and future, such that both ‘now’ and ‘peeled’ are consistent with it. Here, no time shift is required. We found no difference in ERPs between Punctual and Iterative contexts either at ‘now’ or at the second verb. However, reference shifts modulated oscillatory signals. ‘Now’ and the second verb in Punctual contexts resulted in similar responses: an increase in gamma power with a left-anterior distribution. Gamma bursts were absent in Iterative contexts. We propose that gamma oscillations here reflect the binding of temporal variables to the values allowed by constraints introduced by temporal expressions in discourse.

## Introduction

“In the end all she can say is: Oh, but won’t all that be too much trouble, *now*? *Now* being weighted with several troublesome meanings; you may take your choice.”

Alice Munro

Humans are endowed with the cognitive capacity to represent events and move back and forth in mental time [1]. We can recall what has happened in the past, we can consider what could have happened, had the circumstances been different, and we can imagine about what might take place in the future. We are moreover able to refer to past, present and future events. Natural language offers preeminent ways to do so, as well as to induce mental shifting through time. Indeed, it has been proposed that the evolution of mental time travel is intimately connected with the evolution of language [2], and recent work in theoretical linguistics suggests that a cognitive representation of time, different in several crucial ways from physical representations of time, is necessary to account for a range of phenomena in the semantics of tense and aspect [3].

Languages use verbal inflections and phrasal markers, temporal adverbs, prepositions and pronouns, and other lexical and grammatical devices as means for distinguishing and shifting between the times of different episodes [4]. Here, we make use of the temporal adverb ‘now’ to examine mental time shifting. We consider sentences in which the adverb ‘now’ is followed by a past tensed verb, as in ‘now the boy peeled the fruit’. These constructions occur with some frequency in ordinary language, and they are acceptable so long as ‘now’ can refer to a past time frame, which is either specified by a temporal marker in the preceding discourse, or is common knowledge between speaker and hearer. Therefore, we embedded these sentences into narratives, in which ‘now’ either can be anchored to a time frame lying entirely in the past, as in (1), or has to be evaluated relative to a temporally unspecified interval, as in (2):

(1) An hour ago the boy stole candy and now he peeled the fruit. (PC)

(2) The whole afternoon the boy stole candy and now he peeled the fruit. (IC)

Here we call (1) a Punctual context (PC) as the initial noun phrase ‘an hour ago’ specifies a narrow time interval in the past of the moment of speech, in which a punctual or relatively short event can be located. We call (2) an Iterative context (IC) as ‘the whole afternoon’ introduces a sufficiently wide temporal frame as to accommodate repeated occurrences of an event, like stealing candy, which may therefore extend beyond the past, into the present and future. The rationale for using sentences like (1) and (2) as stimuli in our experiment is the following. In PC, the narrative describes events that unfold entirely in the past, until ‘now’ is encountered. We therefore expect a first time shift, from past to present, at the adverb ‘now’ in PC. When the subsequent verb is processed, another shift, this time from present to past, occurs in PC. Because in IC the temporal frame may extend into the present, the narrative time needs not shift either at ‘now’ or at the subsequent past verb. We conducted an EEG study to test two predictions. Firstly, that ‘now’ and the second main verb (e.g., ‘peeled’) leave specific traces on the EEG signal in PC relative to IC. Secondly, that these brain responses are very similar or identical at ‘now’ and at ‘peeled’ in PC, as indeed follows from the notion that the adverb and the subsequent verb trigger identical time shifts with opposite sign: past-to-present at ‘now’, present-to-past at the verb. Below we provide the essential linguistic background on the semantics of ‘now’ and a formal treatment of the above predictions, contrasting three processing models. Finally, we discuss what aspects of EEG signals may be affected by mental time shifting in narratives.

### 1. Linguistic background

Early formal semantic theories assumed that ‘now’ always refers to the time of the utterance, which for the speaker coincides with the present. However, Prior [5] noted that ‘now’ should not *redundantly* refer to the present. That ‘now is the case that *p*’ may not mean the same as ‘it is the case that *p*’ can be seen from numerous natural language examples, e.g., contrast ‘John goes to work by bike’ with ‘Now John goes to work by bike’. Still, Prior suggests, whenever ‘now’ is used, “however oblique the context in which it occurs, the time it indicates is the time of the utterance of the whole sentence” (see also Kamp [6]).

The Prior-Kamp analysis of ‘now’ was radically transformed by later work by Kaplan [7], drawing partly on the intensional theory of indexicals by Bar-Hillel [8]. On this account, ‘now’ has its meaning determined by the *context* of the utterance. The meaning of ‘now’ is such that its referent relative to context C is the time of C. Therefore, if C is the context of the utterance, then ‘now’ refers to the time of utterance. It follows that a sentence such as ‘I am not here now’ may never be uttered truly, despite its acceptable uses in recordings on answering machines. Predelli [9] proposed that the context that fixes the reference of ‘now’ is not the context of the utterance, as Kaplan had suggested, but the *context of interpretation*, that in which the speaker intends the utterance to be understood. In this theory, ‘I am not here now’ means the speaker is not there at the moment the hearer is listening to the message.

Matters are however more complicated. That ‘now’ needs not refer to the present moment (Prior-Kamp), either for the speaker (Kaplan) or for the hearer (Predelli), can be seen in narratives like ‘Napoleon’s troops are now advancing’ [10]. Here, ‘now’ is a link in an anaphoric chain extending before this sentence, to a preceding bit of discourse such as ‘It is May 1812’. The referential anchor may not be explicit, but may be part of common knowledge between the speaker and the hearer. Moreover, there is no need to regard such uses of ‘now’ as part of a make-believe game (see Voltolini [11] for discussion), in which the speaker invites the hearer to pretend those historical events are taking place in the present. Historical uses of ‘now’ merely draw the attention of the audience to a specific point in time, to be taken as the chronological reference. Seen this way, ‘now’ in ‘I am not here now’ refers to any future time at which the listener may hear the recorded message. Most importantly given the aims of the present study, ‘now’ can be used to refer to *imaginary times* in works of fiction, as in this passage from the story Among the Pond People [12]:

(3) There came a time when the Stickleback Father wanted to look fierce, but that was later. Now he went to work to build his nest.

Here, the reader’s mind is first made to travel to a specific interval in a fictional past (‘There came a time…’), and is then guided to an even earlier time frame (‘but that was later’) which is referred to using ‘now’. That is precisely how the adverb is used in our experimental stimuli, (1) and (2). These remarks suggest, at a minimum, that ‘now’ can be used either *indexically*, referring to the present moment of speech, or *anaphorically*, referring to a real or a fictional time interval specified by a noun phrase, as in (1) and (2), or by another anchoring expression in the narrative [13]. Based on the distinction between indexical and anaphoric uses of ‘now’, we can outline three alternative processing theories, that will yield specific predictions for processing PC and IC in our study.

### 2. Processing theories and predictions

We consider the predictions licensed by three processing theories, summarized in Table 1. The first is the Indexical Theory (Table 1, 1). Here, ‘now’ functions as a *pure indexical*, referring to the moment of speech in all contexts. Also, there is no anaphoric processing involved. The reference of each temporal expression is computed locally relative to the constraints set up by the preceding expression. In IC (2), ‘The whole afternoon’ introduces a time frame that is not anchored to the mental timeline: it may be located in the past of the moment of speech, but may extent into the present and future. Indeed, ‘the whole afternoon’ could be followed by a main verb phrase such as ‘I will be busy teaching’. In (2), the first verb ‘stole’ must refer to a past time, which is consistent with the unspecified temporality of the previous noun phrase. This temporal constraint is trivially satisfied, and no reference shift occurs (hence the ‘–’ sign in Table 1). But when ‘now’ is processed, a shift to the present occurs (‘+’ sign). ‘Now’ signals that the events described by upcoming verb phrases should take place in a time interval including the *present*, and when the verb ‘peeled’ is encountered, a second time shift, from present to *past* occurs (Table 1, 1.1). Two analogous reference shifts occur, at ‘now’ and at the second verb, in PC too (Table 1, 1.2). In conclusion, the Indexical Theory predicts no processing difference between Punctual and Iterative constructions.

**Table 1:** Summary of the computations involved in processing the temporal adverb ‘now’ and past-tensed verbs in Iterative and Punctual contexts according to the three theories considered here. Each cell in the table shows the semantic constraint introduced by the temporal expression in the top row: e.g., a simple-past tensed verb requires the event it describes to be located in the past of the moment of speech (E<now). The straight leftward arrow signifies that each constraint is evaluated relative to the previous one, as per the indexical theory. The curved leftward arrow indicates anaphoric processing: each constraint is evaluated relative to the temporal expression specifying the narrative’s time frame, e.g., the sentence-initial noun phrase ‘An hour ago’. The minus sign ('-') indicates no shift in narrative time is required to preserve discourse coherence. The plus sign ('+') indicates a shift is required.

	NP	V_1_	*Now*	V_1_
**Indexical Theory**	←	*E<now*	*E = now*	*E<now*
1.1. Iterative context (IC)	*E*⪓*now*	*−*	*+*	*+*
1.2. Punctual context (PC)	*E<now*	*−*	+	*+*
**2. Anaphoric Theory**	⤺	*E<now*	*E = then*	*E<now*
2.1. Iterative context (IC)	*E*⪓*now*	*−*	*−*	*−*
2.2. Punctual context (PC)	*E<now*	*−*	*−*	*−*
**3. Hybrid Theory**	⤺	*E<now*	*E = now*	*E<now*
3.1. Iterative context (IC)	*E*⪓*now*	*−*	*−*	*−*
3.2. Punctual context (PC)	*E<now*	*−*	+	*+*

The second theory considered here is the Anaphoric Theory (Table 1, 2). Here, all temporal expressions are potentially *anaphoric*, and can thus be bound to discourse elements that provide coherence to the narrative. One such element is the initial noun phrase. A key aspect of the theory is that immediate reference to the discourse model is made. Because interpretation is simultaneous with the construction of a propositional representation [14], there is no early stage at which a default meaning of ‘now’, e.g., its indexical character, is activated and used in interpretation. Because in IC (2) the temporal frame, set up by the initial noun phrase, can include the past, present or future, verbs may be bound to it regardless of their tense, and so can temporal adverbs: ‘now’ here means ‘then’, i.e., whenever the events described in discourse take place (Table 1, 2.1). This applies also if the initial noun phrase refers to a specific past time frame, as in PC (1). The Punctual context is more constraining but still allows all the other temporal expression to be anchored to the initial noun phrase, with no need for reference shifts (Table 1, 2.2). Therefore, the Anaphoric Theory predicts no processing difference between Punctual and Iterative contexts either at ‘now’ or the subsequent verb.

The third option is a Hybrid Theory, which retains elements of both the Indexical and the Anaphoric theories. The first aspect, shared with the Indexical Theory, is that ‘now’ refers to the present moment of speech, immediately and automatically across contexts. However, this indexical meaning is just the initial interpretation, and may be overridden by anaphoric relations, should discourse coherence demand it. In IC (2), the theory predicts no reference shifts to occur: the constraints set up by the initial noun phrase are so relaxed that both verbs, as well as ‘now’, regardless of whether it gets an indexical or anaphoric sense, can be accommodated (Table 1, 3.1). Crucial differences arise in PC (1). Here, the first main verb can be bound to the temporal frame specified by the initial noun phrase, as both lie in the past of the moment of speech. However, ‘now’ cannot, as it refers by default to the moment of the utterance. Without loss of coherence, the narrative time can be shifted to the present, and ‘now’ starts to function as the main discourse element to which verbs should be bound. This sets up a formal constraint that all upcoming verbs should have a present tense form. This requirement is violated by the second main verb, and the coherence of the narrative can only be rescued by *undoing the former reference shift*, through a second time shift, from present to past, which furthermore requires that ‘now’ takes on an anaphoric meaning, instead of its initial indexical sense. Therefore, the Hybrid Theory implies that ‘now’ is more costly to process in Punctual than Iterative contexts, and that so is the second main verb (Table 1, 3.2).

The Hybrid Theory makes no prediction as to the particular neural signals that may be modulated by the reference shifts at ‘now’ and at the verb in PC. The purpose of our study is to search, in event-related potentials (ERPs) and oscillations, for any effects that can be compared to the theoretical pattern. What the theory does predict, however, is that the two shifts in narrative time leave very similar traces on brain signals. In PC, ‘now’ shifts the narrative time from past to present, and the verb moves it back to the past. This second shift implies that the meaning of ‘now’ is recomputed, so that it can be anaphorically bound to the time of the initial noun phrase. In the Hybrid Theory, temporal expressions can behave anaphorically, should their default meaning compromise discourse coherence. The Hybrid and the Anaphoric theories differ only in that the former assumes the indexical meaning of ‘now’ is activated in the first instance in processing, whereas in the latter account the meaning of ‘now’ is immediately determined by discourse. Our experiment can therefore be seen as a direct test of the notion that ‘now’ has a default indexical meaning (i.e., its Kaplanian character) that is automatically, albeit tentatively, activated during processing. Before we turn to a description of the methods of our experiment, we consider the possible brain signatures of narrative time shifts, based on the recent neuroscience literature.

### 3. Neural correlates of reference shifts

We consider two signal domains, event-related brain potentials (or ERPs) and oscillations. Traditionally, in psycho- and neurolinguistics, language processing has been investigated in situations in which expressions take default meanings. Research on the neural effects of *violations* of default semantic and grammatical constraints has provided valuable insights into sentence processing (see [15–17] and ensuing research). However, a potentially much broader range of linguistic phenomena involving non-default meanings, for instance in sense and reference shifts, still remains to be systematically investigated (see [18–21] for some work in this direction). This is partly due to the proven efficacy and diffusion of experimental designs using linguistic anomalies, and partly to the assumption that the neural effects of subtler processes such as sense or reference shifts may be too weak to detect. As we argue here, also based on the results of the present experiment, this sensitivity concern may apply especially to ERPs.

At least two ERP waves are modulated by linguistic factors: the N400 [15] and the P600 [16–17]. These have been often, but not unanimously, viewed as signatures of lexical semantic and grammatical processing, respectively, as their amplitudes relative to control stimuli are negatively correlated with the degree of semantic or grammatical fit (their amplitudes are positively correlated with processing effort) of the eliciting word given the preceding sentence or discourse context. ‘Degree of fit’ here reflects the statistics of the linguistic ‘corpus’ that speakers have been exposed to throughout their life, which can, however, be overridden by local contextual dependencies among words (e.g., in fictional discourse; see [22]). If default lexical semantic and grammatical constraints are coded in the statistics of the brain’s linguistic corpus, departures from the most likely patterns will result in proportional increases of the N400 or P600 amplitudes. The N400 and P600 are robust markers that are routinely used as dependent variables in research on semantic and grammatical processing, as well as reference-time violations [23–24]. Nonetheless, the N400 and P600 may not necessarily be selective or responsive to sense or reference shifts, and this may be the case especially if such shifts are freely occurring in spoken and written language, and are therefore well represented in the brain’s linguistic corpus.

In the present experiment, intermixed with critical sentences inducing time shifts such as (1) and (2), we included sentences containing semantic or grammatical anomalies, as well as matched control stimuli, to assess whether our data have sufficient signal and statistical power to produce N400 or P600 effects in standard elicitation conditions. In this way, a lack of ERP effects in critical sentences can be taken as evidence that time shifts in narratives fail to modulate time-locked responses. Consequently, we analyzed our EEG data for changes in power in specific frequency bands over time, to assess whether the presence or lack of ERP effects is accompanied by specific oscillatory responses to time shifts in narratives. We expect power changes in the theta (4–7 Hz) or in the gamma (30Hz and higher) bands, which have been associated with memory retrieval and integration operations in language [25].

More specifically, several studies reported power modulations in the theta and gamma frequency bands in semantic processing tasks. Braeutigam et al. [26] observed transient gamma oscillations at intermediate (300 ms) and at long (500 ms) latencies for incongruent words, thus extending beyond the time window at which the N400 typically occurs. Hald et al. [27] report increased power in the theta band in an interval of 300–800 ms following the onset of the critical words, largest over mid-frontal regions for semantic violations. In the gamma band, however, power increased for correct sentences, and this effect was absent following semantic incongruities. Therefore, and most relevant for the present study, gamma activity may reflect the normal process of semantic integration of words into a sentence or discourse, rather than a brain response to an anomalous stimulus. This conclusion is also supported by later findings. For example, Penolazzi et al. [28] reported effects of sentence correctness in the gamma frequency range (30–100 Hz), with well-formed sentences showing higher gamma power than anomalous sentences.

Based on previous experiments, we can therefore ground the processing predictions above in neural dependent variables, as follows. We expect that the time shifts induced by ‘now’ and by the second verb in PC relative to IC should modulate either the amplitude of the N400 evoked by these words, or power in the gamma frequency band. Crucially, brain responses at these two positions in PC should be very similar, in terms of latency, topographical distribution and frequency range. This follows from the fact that the past-to-present shift at the adverb ‘now’ and the present-to-past shift at the subsequent verb are instances of the same type of computation (see above and Table 1).

## Methods

All experiments described below were conducted using Dutch stimuli and participants. Examples of critical sentences in Dutch, alongside their English translations, as described in the Introduction, are given below:

Punctual context (PC):

(4) Een uur geleden pikte de jongen een snoepje en nu schilde hij de vrucht.

[An hour ago stole the boy a candy and now peeled he the fruit.]

An hour ago the boy stole a candy and now he peeled the fruit.

(5) Een uur geleden brak de man het glas en nu tapte hij het bier.

[An hour ago broke the man the glass and now poured he the beer.]

An hour ago the man broke the glass and now he poured the beer.

Iterative context (IC):

(6) De hele middag pikte de jongen snoepjes en nu schilde hij de vrucht.

[The whole afternoon stole the boy candies and now peeled he the fruit.]

The entire afternoon the son stole candy and now he peeled the fruit.

(7) De hele middag brak de man glazen en nu tapte hij het bier.

[The whole afternoon broke the man the glass and now poured he the beer.]

The entire afternoon the man broke glasses and now he poured the beer.

### 1. Acceptability ratings

Acceptability ratings on a five-point Likert scale (1 very unacceptable; 5 very acceptable) were collected from 45 undergraduate students. None of them took part in the subsequent self-paced reading experiment or EEG experiment. The pretest was administered as an online questionnaire and comprised 50 critical sentences (25 PC and 25 IC) and 77 filler sentences. Participants could take as much time as needed to rate the sentences. The results show that PC and IC sentences are rated as equally acceptable, with mean scores 2.282 (SD = 1.310) and 2.282 (SD = 1.306) respectively. This is an indication that any processing differences that may be found between the two sentence types cannot be attributed to differences in their off-line appreciation.

### 2. Self-paced reading experiment

#### 2.1. Participants

Eighteen native speakers of Dutch participated in a self-paced reading study (12 female; mean age 23.22 years; age range 18–29 years). Participants were evenly assigned to two different lists, which contained either the PC or the IC version of each sentence, to avoid repetition effects. All participants received university education; none studied linguistics. All participants had corrected-to-normal or normal visual acuity. They were unaware of the purpose of the experiment and they received €3 each as a compensation for a single 20-minute testing session. Participants signed an informed consent drafted according to the declaration of Helsinki before the experiment commenced.

#### 2.2. Materials

Each participant read 50 critical sentences in Dutch: 25 PC and 25 IC. Sentences had the syntactic structure of examples (4)-(7), but used different lexical items. We included a first set of 39 fillers, of which 13 contained a semantic violation as in (8), and were expected to elicit an N400, and 26 correct counterparts, all adapted from [29]:

(8) *De prooi werd door de hongerige leeuwen **opgeschreven** terwijl de peuter angstig toekeek.

[The prey was by the hungry lions written-down while the toddler frightened watched.]

*The prey was written down by the hungry lions while the toddler watched*, *frightened*.

The stimuli comprised a second set of 39 fillers, with 13 grammatical violations as in (9), which were expected to elicit a P600 effect, and 26 correct counterparts, all adapted from [30]:

(9) *Ze heeft alleen haar beste vriendin **uitnodigen** zonder aan mij te denken.

[She has only her best friend invite-INF without of me to think.]

*She has only inviting her best friend without thinking of me*.

Finally, the stimuli included 25 filler sentences with the syntactic structure of (4)–(7) but containing a present tensed verb in the first clause instead, and 40 unrelated filler sentences with varying structure and content. The present tensed sentences were included to render it impossible for participants to anticipate the temporal reference of the second main verb, which would presumably reduce or prevent any effects occurring after word onset.

#### 2.3. Apparatus and procedure

The stimuli were presented using E-Prime (Psychology Software Tools Inc., 2001). The experiment took place in a dimly lit and sound-attenuating room. Participants were tested in individual sessions and were seated at about 80 cm distance from the computer screen. Sentences were presented visually, phrase-by-phrase. Each phrase consisted of 1, 2 or 3 words and was presented in the center of the screen, in white on a black background, using Verdana font size 18. Each trial started with a centered row of five X’s to mark the fixation spot. The participant started reading the sentence and moved to the next phrase by pressing the space bar. Reaction times from the onset of the phrase to the pressing of the space bar were recorded and were used as the dependent measure in this experiment.

Participants were instructed to read the sentences at their own pace and were asked to respond to a word that appeared after the sentence: by pressing the z-key or the /-key on a QWERTY-keyboard, participants had to indicate whether the word was semantically related to the previous sentence or not. Participants were also instructed to count the number of errors they detected in the sentences during the course of the experiment, of which they had to give an approximate account at the end of the experiment. This additional task had the purpose of checking whether participants attended to the sentences. Based on this, no participants were excluded from the final analysis.

#### 2.4. Statistical analyses

An ANOVA model with factors Condition (PC or IC) and Sentence Position (7 levels: the first clause verb, the first clause subject, the conjunction ‘and’, the indexical ‘now’, the critical verb, the second clause subject, and the second clause object), and Reading Time as the dependent measure, was used.

### 3. EEG experiment

#### 3.1. Participants

Thirty-two native speakers of Dutch participated in the EEG study. None of them had participated in the self-paced reading study. Five participants were excluded from further analyses because EEG trials contaminated by artifacts were more than 20% of the total number of trials, or because the participant could not finish the test. The remaining 27 participants (18 female; mean age 21.63 years) were evenly distributed over lists. All participants were medical students, and were right-handed with normal or corrected-to-normal vision. Participants were unaware of the purpose of the study and received €18 for a single two-hour session. Each participant signed an informed consent based on the declaration of Helsinki before the experiment commenced. The study was approved by the Neuroimaging Center at the University of Groningen.

#### 3.2. Materials

The stimulus sentences were the same as those used in the self-paced reading experiment (see 2.2).

#### 3.3. Apparatus and procedure

The equipment and procedure were similar to those of the self-paced reading experiment, except that stimulus presentation was word-by-word in the EEG experiment, and not phrase-by-phrase. Each word was presented for 240 ms, followed by a blank screen of 240 ms. At the end of each trial participants had to indicate whether the sentence they had just read was acceptable or not, by pressing the z-key or the /-key on a QWERTY-keyboard. Participants were instructed to respond immediately after the end of the sentence, which was signaled by a full stop following the final word. Participants were instructed to blink only during the 500 ms appearance of the five X’s initiating each sentence.

#### 3.4. Behavioral Data Analyses

Behavioral responses in the EEG study were analyzed using an ANOVA model with factor Condition (PC or IC) and the proportion of acceptable judgments of the sentences as the dependent variable.

#### 3.5. EEG recordings

The EEG was recorded from 64 tin electrodes mounted in an elastic cap (Electro-Cap International Inc.) and arranged according to the extended international 10–20 system. Two additional electrodes were placed on the left and right mastoids. The electrode on the left mastoid served as the reference during the recording. The EOG was recorded using four bipolar tin electrodes situated on the outer canthi of each eye and above and below the left eye. The data were digitized using a Refa8–64 amplifier (TMS International) with a 500 Hz sampling rate, a low pass filter at 140 Hz, and a 10 s time constant. The ground electrode was mounted on the sternum.

#### 3.6. Event-related potentials

Data were analyzed using FieldTrip [31]. Segments were extracted from the EEG from each channel starting 200 ms before and ending 1000 ms after the onset of each critical word (‘now’, and the second verb), and were baseline-corrected using the 200 ms pre-stimulus interval. All channels were then re-referenced off-line to a linked mastoid. Artifact identification and rejection were carried out as follows: (1) trials containing activity exceeding a ±200 μV threshold were detected; (2) trials containing muscle artifacts were identified by thresholding the *z*-transformed values of the 100–125 Hz band-pass filtered EEG; (3) segments containing eye movements were identified by thresholding the *z*-transformed values of the 1–15 Hz band-pass filtered EOG. Steps (2)-(3) detect high-amplitude signal changes in specific frequencies in each channel as follows: (i) the amplitude of the signal over time (the Hilbert envelope) is computed; (ii) its mean and standard deviation are computed; (iii) every time point is *z*-normalized (i.e., its mean is subtracted and divided by the standard deviation); (iv) *z*-values are averaged per time point. Trials selected in steps (1)-(3) were discarded. On average 99.6% of trials (SD = 0.28%) survived artifact rejection. Finally, the data were low-pass filtered at 30 Hz.

**Fig 1:**
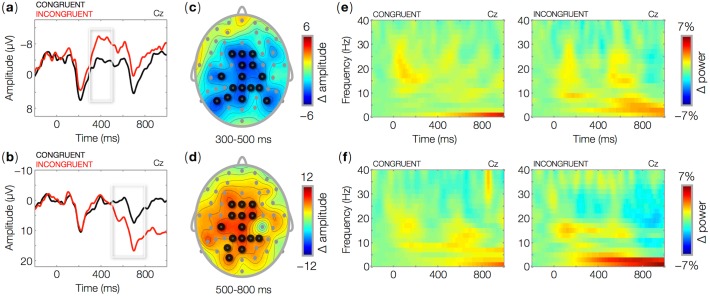
ERPs time locked to the onset (0 ms) of critical words in semantically (top row) and grammatically (bottom row) congruent and incongruent sentences. Waveforms show ERPs for congruent and incongruent trials at the central-midline electrode Cz (a-b). Topographic maps show the mean amplitude difference between incongruent and congruent trials in the 300–500 ms (c) and 500–800 ms (d) time intervals relative to visual word onset (0 ms). Wavelet-based time-frequency representations (TFR) of EEG data for critical words in semantically (e) and grammatically (f) congruent and incongruent sentences at the central-midline electrode Cz. The plots show percent power changes relative to baseline as a function of time and frequency.

**Fig 2:**
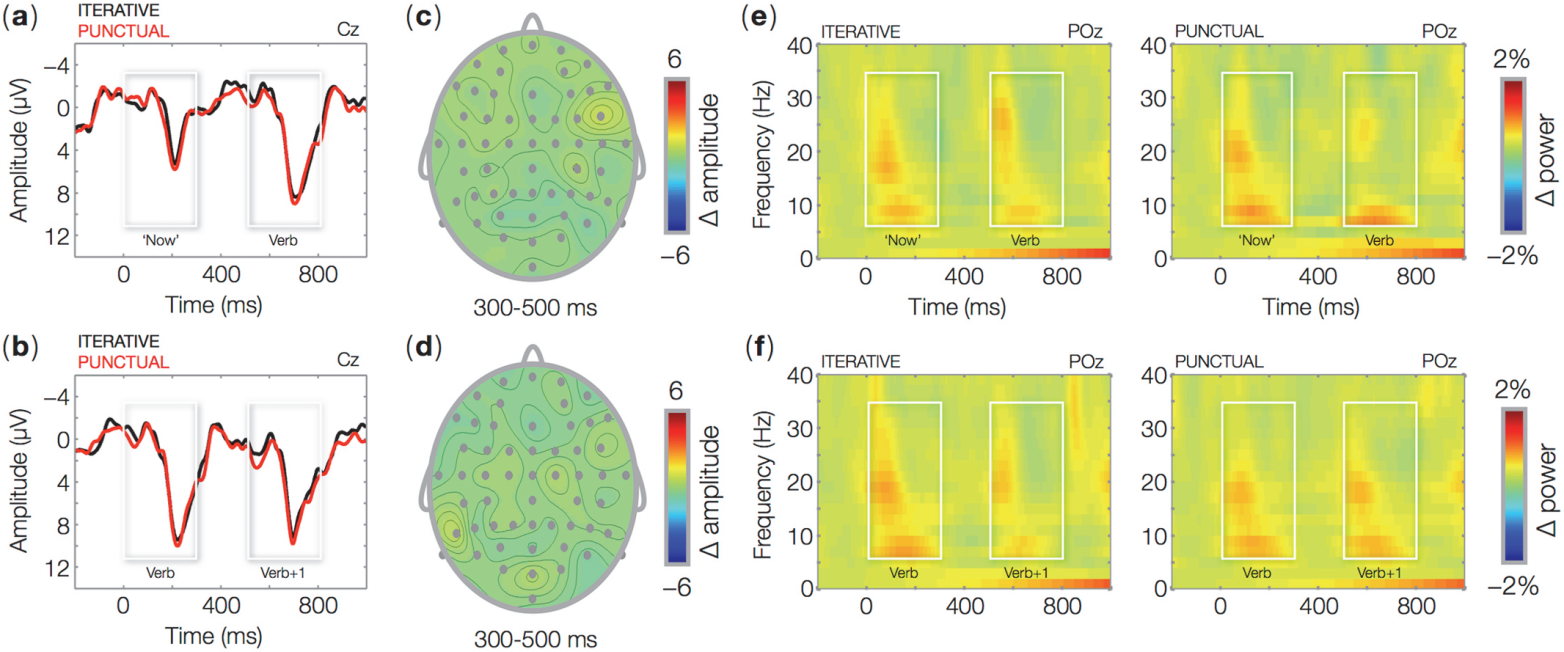
ERPs time locked to the onset (0 ms) of the word 'now' (top row) and of the second main verb (bottom row) in Iterative and Punctual contexts. Waveforms show ERPs for Iterative and Punctual trials at the central-midline electrode Cz (a-b). Topographic maps show the mean amplitude difference between Punctual and Iterative trials in the 300–500 ms time interval relative to visual word onset (0 ms). Wavelet-based time-frequency representations (TFR) of EEG data for 'now' (e) for the second main verb (f) in Iterative and Punctual contexts at the parieto-occipital midline electrode POz. The plots show percent power changes relative to baseline as a function of time and frequency.

ERPs were obtained by averaging over artifact-free segments in each condition for each participant separately. Grand-averages over participants were used to produce Figs. 1–2. ERP data were statistically analyzed by means of a cluster-based permutation procedure [32] with the following steps. A sample is defined as a (time, electrode) pair. For N400 and P600 sentences, and for PC and IC sentences, the two experimental conditions (Incongruent/Congruent; Punctual/Iterative) were compared by means of a dependent-samples *t*-test on each sample. Samples for which the *t*-tests yielded a *p*<0.05, and that were moreover connected in space (neighboring electrodes) and in time (adjacent points), were clustered together, and the sum of *t*-values from sample-level *t*-tests in the cluster was used as a cluster-level *t*-value (*tsum*). The sample-level alpha threshold was therefore 0.05. The minimum number of neighboring electrodes that would count as a cluster was 2. Two electrodes are considered neighbors if they can be connected by an imaginary path that does not cross the paths connecting each of them with any other of their neighbors. The cluster-level *p*-value, whose alpha was also 0.05, was estimated separately by means of a Monte Carlo simulation: ERP averages in each spatio-temporal cluster for each participant from both conditions were collected in a single set; the resulting set was randomly partitioned into two subsets of equal size, and a *t*-test was used to compare the means of the subsets; these steps were repeated 1000 times, and the cluster-level *p*-values were estimated as the proportion of random partitions that resulted in a larger *t*-statistics than the one on the observed ERP averages.

#### 3.7. Time-frequency representations

Time-frequency representations (TFRs) were computed separately for each condition (Iterative context or IC, and Punctual context or PC) relative to the onset of ‘now’ and of the critical verb. Mean power changes over time across frequency bands were quantified in each condition relative to baseline. EEG segments relative to the onset of ‘now’ and of the verb of PC and IC sentences were preprocessed using the same procedure used for ERP analyses, with the following differences: the pre-stimulus interval was 500 ms, which was also used for baseline correction, and epochs ended 1500 ms after word onset. No low-pass filter was applied to the data. Artifact identification and rejection criteria were as described in 3.6. TFRs were computed by means of Morlet wavelet transforms [33]. Morlet wavelets were obtained by composing a carrier wave in each frequency step with a Gaussian envelope. The width in cycles of the resulting wavelet was 7. The length of the wavelet was 3 standard deviations of the implicit Gaussian kernel. TFRs for each EEG segment were derived as the squared norm of the convolution of the Morlet wavelets with the EEG time series [34]. TFRs were computed for frequencies ranging from 1 to 120 Hz in steps of 2 Hz. TFRs were averaged over trials for each participant in each condition. To normalize for individual differences in EEG power, and for differences in absolute power between frequency bands, average power values were expressed as percent change relative to power in a baseline interval from-500 to 0 ms prior to critical word onset.

**Fig 3:**
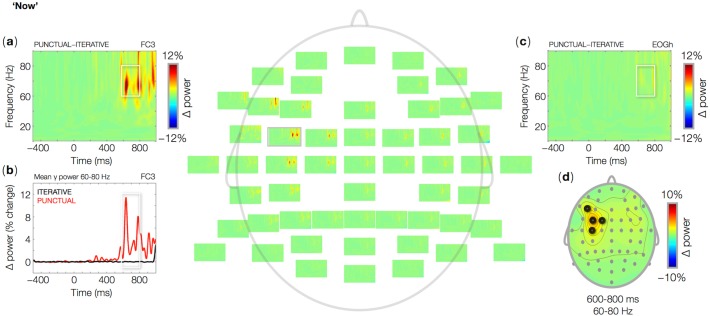
Wavelet-based time-frequency representations (TFR) of EEG data at ‘now’ in critical sentences. Single-channel plots show percent power changes relative to baseline as a function of time and frequency for the mean power difference between Punctual and Iterative trials. The data for a single channel (FC3) in which the effect was largest is shown in (a), and the time course of the effect in the 60–80 Hz frequency band is shown in (b). Absence of mean power differences between conditions in the horizontal eye channel is shown in (c). The topographic map (d) shows the mean power difference between Punctual and Iterative trials in the 60–80 Hz frequency band, in the 600–800 ms time interval relative to visual word onset (0 ms). Black circles represent electrodes belonging to a statistically significant cluster throughout the time interval of interest (600–800 ms).

**Fig 4:**
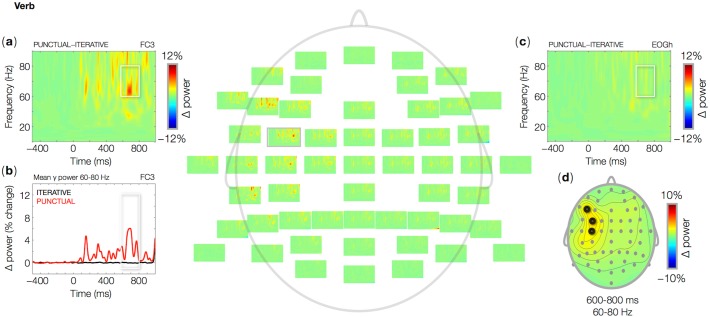
Wavelet-based time-frequency representations (TFR) of EEG data at the second main verb in critical sentences. Single-channel plots show percent power changes relative to baseline as a function of time and frequency for the mean power difference between Punctual and Iterative trials. The data for a single channel (FC3) in which the effect was largest is shown in (a), and the time course of the effect in the 60–80 Hz frequency band is shown in (b). Absence of mean power differences between conditions in the horizontal eye channel is shown in (c). The topographic map (d) shows the mean power difference between Punctual and Iterative trials in the 60–80 Hz frequency band, in the 600–800 ms time interval relative to visual word onset (0 ms). Black circles represent electrodes belonging to a statistically significant cluster throughout the time interval of interest (600–800 ms).

Statistical analyses of TFR data used the cluster-based permutation approach described above. A sample is a (time, frequency, electrode) triple. Experimental conditions are compared at each sample using a dependent samples *t*-test. The rest of the procedure is as described in 3.6. To further analyze statistically the reported TFR effects, we employed generalized linear models (GLMs) using mean power from anterior channels bilaterally (F8, F3, F4, F5, F6, F7, FC3, FC4, FC5, FC6) in the frequency bands and time intervals of interest (Figs. 3–4) as a dependent variable, and Iterative or Punctual context as the predictors. The GLM was computed using a Gaussian variance function and an identity link function.

## Results

### 1. Self-paced reading experiment

Reading a phrase on average took 485.7 ms (SD = 435.36 ms) in PC and 484.94 ms (SD = 454.96 ms) in IC. The ANOVA showed no main effect of Condition and no interaction with Sentence Position.

### 2. EEG experiment

#### 2.1. Behavioral measures

Participants judged PC sentences to be acceptable in 55.26% (SD = 36.31%) of the trials, and IC sentences in 53.93% (SD = 34.42%) of the trials. There was no main effect of the factor Condition in the ANOVA (*F*(1,23) = 0.5, *p =* 0.486). These data suggest participants judged the sentences to be equally acceptable. This is in line with the results of the acceptability ratings study.

#### 2.2. Event-related potentials

Sentences that typically induce N400 and P600 effects were included in the present experiment as a check for the statistical power afforded by our data. Semantically incongruent sentences indeed produced a larger N400 than did congruent sentences. The effect started around 300 ms, peaked at 400 ms, and was reduced at 600 ms relative to word onset (Fig. 1A; Table 2, 1.1). Consistent with previous research, the observed N400 had a central-posterior distribution (Fig. 1C). Grammatical anomalies elicited a larger P600 compared to congruent sentences. Consistent with earlier work, the P600 appeared around 500 ms and peaked at approximately 700 ms (Fig. 1B; Table 2, 1.2). The effect had a central-posterior distribution (Fig. 1D). These effects show that the present experiment had sufficient statistical power to reveal neural correlates of incongruities of meaning and grammar (see [35] for a thorough analysis of these effects). There were no differences in ERP amplitudes between PC and IC either at ‘now’, at the second verb, or at the sentence-final word (Fig. 2A-D; Table 2, 2.1–2.3). However, effects were observed as modulations of oscillatory brain responses.

**Table 2:** Results of cluster-based permutation statistics on ERP data comparing incongruent and congruent critical words in grammatically and semantically anomalous sentences, and the word occupying the ‘now’-, verb- and sentence-final positions in critical sentences following Punctual and Iterative contexts. The sum of t-statistics in the largest cluster, the p-value of the effect in that cluster, and cluster size in number of (time, channel) samples are shown.

**1**	**N400/P600 fillers**	**Positive clusters**	**Negative clusters**
1.1	Semantic anomalies	*t_sum_* = 69, *p* = 0.67, S = 28	*t_sum_* = −4480.2, *p* = 0.007, S = 1301
1.2	Grammatical anomalies	*t_sum_* = 12227, *p*<0.001, *S* = 3409	*t_sum_* = −123.971, *p* = 0.523, *S* = 49
**2**	**Punctual/Iterative**	**Positive clusters**	**Negative clusters**
2.1	*Now* position	*t_sum_* = 108.158, *p* = 0.557, S = 44	*t_sum_* = −243.215, *p* = 0.295, S = 80
2.2	Verb position	*t_sum_* = 287.612, *p* = 0.198, *S* = 100	*t_sum_* = −81.247, *p* = 0.633, *S* = 32
2.3	Sentence-final position	*t_sum_* = 310.945, *p* = 0.252, S = 126	*t_sum_* = −8.375, *p* = 0.93, S = 4

#### 2.3. Time frequency analyses

In N400 stimuli, we found no clear modulation of power in any frequency band. The seeming power increase in the theta frequency range (Fig. 1E) for incongruent items is not statistically significant (no clusters were found). We did find, however, a power increase in the delta (1–4 Hz) frequency range in grammatically incongruent stimuli (Fig. 1F).

At frequencies up to 40 Hz, PC and IC result in very similar responses both at ‘now’ and at the subsequent verb. Power increases relative to baseline can be observed in the theta (6–8 Hz) and beta (16 Hz and up) bands (Fig. 2E-F), corresponding to the exogenous ERP components N1 and P2 evoked by every visual word. In this frequency range, no differences between PC and IC were observed (no significant clusters), while we did find effects of PC as compared to IC in the gamma band (Figs. 3–4). In IC, we found no gamma power changes relative to baseline, either at ‘now’ or at the second verb. The temporal adverb ‘now’ in PC induced a surge of gamma power (60–80 Hz) between 600 and 800 ms from word onset relative to IC (Fig. 3A-B; Table 3, 1). The effect was largest over left-anterior recording sites (Fig. 3D). The horizontal eye channel did not show any effects in the same frequency range (Fig. 3C). As is the case at ‘now’, the second verb in IC induced no significant power changes relative to baseline in any frequency band, but in PC it induced a gamma burst (60–80 Hz) between 600 and 800 ms from word onset (Fig. 4A-B; Table 3, 2), again with a left-anterior distribution (Fig. 4D). There were no changes in gamma power in the horizontal eye channel (Fig. 4C).

**Table 3:** Results of cluster-based permutation statistics (CBP) and generalized linear model statistics (GLM) on time-frequency representation (TFR) data comparing the word ‘now’ and the verb in critical sentences following Punctual and Iterative contexts. For CBP statistics, the sum of t-statistics in the largest cluster, the p-value of the effect in that cluster, and cluster size in number of time, channel, frequency) samples are shown. For GLM statistics, the t-statistic for Punctual or Iterative context as a predictor of gamma-band power and its p-value are reported. The specified time intervals and frequency bands are as in Figs. 3–4.

		CBP statistics: Punctual *vs* Iterative	GLM statistics: Punctual context	GLM statistics: Iterative context
1	*Now* position (Time: 600–800 ms, Frequency: 60–80 Hz)	*t_sum_* = 798.09, *p* = 0.012, S = 535	*t* = 3.145, *p* = 0.002	*t* = 0.017, *p* = 0.986
2	Verb position (Time: 600–800 ms, Frequency: 60–80 Hz)	*t_sum_* = 614.372, *p* = 0.018, S = 419	*t* = 3.296, *p* = 0.001	*t* = −0.044, *p* = 0.965

## Discussion

The temporal adverb ‘now’ has been subject to much theoretical research in linguistics. In particular, it is now established that a purely indexical account, where ‘now’ always refers to the moment of speech, or to the present time for the hearer, does not explain a range of uses of the temporal adverb, such as in historical or narrative contexts like (3). Discourse appears to play a crucial role in determining the time interval to which ‘now’ may refer in each case (besides the literature reviewed in the Introduction, see [36–39]). However, there remains a possibility that, during processing, ‘now’ is initially and tentatively given its default indexical meaning, which can be subsequently recomputed should discourse coherence demand it, based on the available anaphoric antecedents. This is precisely the assumption made by the Hybrid Theory presented above, and contrasted with a purely Indexical Theory and a purely Anaphoric Theory of processing ‘now’ and other temporal expressions in context. Whereas the latter two theories do not entail differences between PC and IC, the Hybrid Theory predicts specific time shifts to occur at ‘now’ and at the subsequent verb in PC relative to IC. In the current study, we aimed at establishing a neural correlate of time shifting in narratives. By analyzing oscillatory brain responses at ‘now’ and at the second verb, we found a pattern that closely matches the key predictions of the Hybrid Theory. We observed nearly identical increases in gamma power in PC relative to IC, at ‘now’ and at the verb. These gamma oscillations are not the product of microsaccades, because they were absent from the horizontal eye channel. Also, they occur in a narrow range of the gamma band, whereas gamma oscillations that originate in eye movements typically have a more broadband distribution [40–41]. We are therefore inclined to regard the observed gamma oscillations as a neural signature of the cognitive operations involved in shifting the time of the narrative given the discourse constraints. These operations bind the variables introduced by temporal expressions to values that preserve discourse coherence (Table 1) [3, 42–43].

Our rating data suggest that Punctual and Iterative sentences are judged as being equally acceptable. This result is consistent with the lack of differences in reading times, as well as a lack of an N400 or a P600 effect at ‘now’ and at the subsequent verb. In contrast to the absence of ERP effects in critical items, N400 and P600 effects were clearly visible in the filler sentences, and were moreover statistically robust. Had ‘now’ or the second past tensed verb been perceived as semantically or grammatically anomalous in PC relative to IC, or vice versa, an N400 or a P600 effect, or some other modulation of ERP components, may have been observed. Given the ERP effects in the filler sentences, we see the absence of an N400 or a P600 in critical sentences as further evidence that Punctual and Iterative contexts are perceived as semantically and grammatically well-formed.

ERP effects are routinely used as dependent measures of the costs of integrating a word in a sentence or discourse context [15–17]. If a word does not satisfy the semantic or grammatical constraints set up by the sentence context, ERP effects such as the N400 and P600 are often observed. However, in some cases contextual constraints can be satisfied by forcing the relevant words to take a different meaning than their default sense: ‘now’ is a case in point, as it can be made to refer indexically to the time of utterance, or anaphorically to a time specified by discourse. Our results show that ERPs may not be sensitive to such flexible forms of constraint satisfaction in discourse processing, and that instead those operations may be reflected in oscillatory brain dynamics.

Behaviorally, no difference was found in the grammatical appreciation or in processing of Punctual and Iterative sentences. However, power analyses of EEG signals did yield processing asymmetries between conditions. We had no prior expectations concerning whether any neural signatures of time shifts would be found in the time-locked domain, in oscillations, or in both. We did, however, predict there would be processing costs for Punctual as compared to Iterative contexts. Specifically, we predicted that the effect that would occur at the verb following ‘now’ would be very similar to the effect found at ‘now’, as the two words induce computationally analogous shifts in temporal reference: past-to-present at ‘now’, present-to-past at the verb. In our own EEG data, this computational similarity is reflected by the strict resemblance of gamma bursts in brain oscillations at ‘now’ and at the verb in PC relative to IC. These gamma bursts peak at approximately the same time post-onset of the two critical words and within the same frequency band. Besides, both effects are most pronounced over left-anterior scalp sites.

The key difference between Punctual and Iterative contexts is that, in the former but not in the latter, temporal restrictions are put on the interpretation of the adverb ‘now’. In the context of the noun phrase ‘an hour ago’, ‘now’ may be used with its default meaning of utterance time, but only at the additional cost of shifting the narrative time to the present. This is reflected in the gamma burst induced by ‘now’. In Punctual contexts, coherence can be preserved only if upcoming verbs describe events in the present tense. Thus, when a past tensed verb comes in, a second shift occurs, that moves back the narrative time to the past. This is reflected in the gamma surge induced by the second main verb in PC sentences. As the two time shifts are formally similar, and the two gamma effects they induce are similar as well, we tentatively view gamma oscillations here as a neural correlate of time shifts in narrative language. More specifically, in line with the constraint-satisfaction framework used to formulate the theories considered here [3,42–43], we argue these gamma bursts may reflect operations that bind temporal variables to the values allowed by the constraints set up by temporal expressions in the preceding discourse. In previous experiments, we found that a failure to satisfy temporal constraints, as in the tense violations at the verb in ‘Last week Vincent paints his house’, leads to an rapid ERP response over left-anterior sites [43], thus with a similar topography as gamma bursts in the present study, as well as to later P600 effects [23–24]. Recomputing the ongoing temporal or causal profiles of discourse representations leads to sustained ERP responses, which emerge in a time window following that of the N400, thus similar to the time course of the gamma modulations reported here [44]. Taken together, these findings suggest at a minimum that temporal semantics recruits different brain networks and neural processes than does the integration of lexical meanings in context, resulting in either earlier or later ERP effects than the N400, and in modulations of other signal domains in the EEG than ERPs, such as gamma oscillations. Gamma oscillations have been associated to binding of feature sets in perception and cognition [45–48], as well as to forms of semantic processing [25–28]. The results of our study can thus be linked up to the view that gamma oscillations reflect general and widespread binding mechanism in the cortex.

This study employed linguistic stimuli to induce time shifts. However, the observed effects in the gamma band should not be taken to be exclusively reflective of linguistic processes. In our experiments, temporal expressions in natural language acted as the carriers of information inducing restructuring effects on the mental representation of events. Shifts in reference time can be induced by language, as well as by other means, such as mental imagery (see [2] for a discussion of relations between language and mental time travel). This is not surprising, as language is not a stand-alone cognitive module, but shares neural resources with other brain systems. The same could be argued about ERP components such as the N400 and P600, which, although sensitive to linguistic constraints, are not specific to language [49–50]. Compared to anomalies of meaning and grammar, the present experiment constitutes an example of the study of subtler yet presumably more widespread neural and cognitive operations involved in processing meaning [42,51–52]. Time-frequency analyses of the EEG can capture aspects of reference shifts for which, in the current study, no ERP counterparts were found. Oscillations may constitute a valuable signal domain for identifying the neural mechanisms of a range of linguistic and cognitive phenomena that may incur in processing costs without necessarily violating statistical regularities or formal constraints.
